# Diphenylamino-Modified Neutral Pt(II) Complexes: Their Aggregation-Induced Phosphorescent Emission and Picric Acid-Sensing Properties

**DOI:** 10.3390/ma17174366

**Published:** 2024-09-03

**Authors:** Qinglong Zhang, Yingying Yan, Rui Cai, Xiao-Na Li, Chun Liu

**Affiliations:** 1 State Key Laboratory of Fine Chemicals, Frontier Science Center for Smart Materials, School of Chemical Engineering, Dalian University of Technology, Linggong Road 2, Dalian 116024, China; zhangqinglong@mail.dlut.edu.cn (Q.Z.); yanyingying@mail.dlut.edu.cn (Y.Y.); cairui@dlut.edu.cn (R.C.); 2 School of Environmental Science and Technology, Key Laboratory of Industrial Ecology and Environmental Engineering (MOE), Dalian University of Technology, Dalian 116024, China

**Keywords:** neutral platinum(II) complex, diphenylamino group, AIPE property, picric acid, aqueous media

## Abstract

Three neutral Pt(II) complexes with diphenylamino-modified 2-phenylpyridine derivatives as cyclometalating ligands and acetylacetone as the ancillary ligand exhibit aggregation-induced phosphorescent emission (AIPE) properties in THF/H_2_O. The crystal structures of the complexes highlight the contributions of non-covalent Pt···Pt interactions and hydrogen bonds to the AIPE properties. These AIPE-active Pt(II) complexes **1**–**3** have been successfully applied to detect picric acid (PA) in aqueous media, affording the lowest limit of detection at 70 nM. Furthermore, three Pt(II) complexes are able to detect PA in common water samples. The quenching of luminescence in the detection can be attributed to photo-induced electron transfer.

## 1. Introduction

Square-planar cyclometalated Pt(II) complexes have garnered considerable attention due to their ability to generate non-covalent Pt···Pt and π-π stacking interactions [1,2,3]. These interactions lead to the aggregation of the molecules, resulting in a remarkable photophysical phenomenon known as aggregation-induced phosphorescent emission (AIPE) and changes in the excited state properties of the corresponding compounds [4,5,6]. For instance, Yam and co-workers reported the solvatochromism and enhanced emission of the Pt(II) complexes possessing terpyridyl ligands for the construction of Pt···Pt and π-π interactions [7]. Subsequently, they reported the formation of various superstructures (including nanorods, nanospheres, nanowires, and nanoleaves) through the self-assembly processes of Pt(II) complexes via molecular engineering [8].

The diphenylamino (DPA) group is a unique structural motif for constructing complexes due to its strong electron-donating nature [9,10,11,12,13]. Introducing the DPA group into a molecule has been proven to be an efficient method for tuning the luminescent properties of phosphorescent materials [14,15,16,17]. In 2019, Sun and co-workers reported an AIPE-active DPA-modified Pt(II) complex, and the non-doped OLED on the basis of this complex exhibited highly efficient deep-red/NIR emission [18]. Recently, we found that incorporating a DPA group onto the cyclometalating ligand of Ir(III) complexes significantly improved their luminescent properties [19,20].

Picric acid (PA) is a potential carcinogen and poses a superior threat to human life, even at low concentrations [21,22]. Therefore, it is increasingly crucial to exploit highly sensitive and selective detection methods for PA. Currently, the systems of most reported Pt(II) complexes to detect PA primarily focus on organic solvents, which greatly limits their practical applications [23,24,25,26,27,28]. Thus, it is essential to establish novel, efficient approaches to detect PA in aqueous media.

**Scheme 1:**
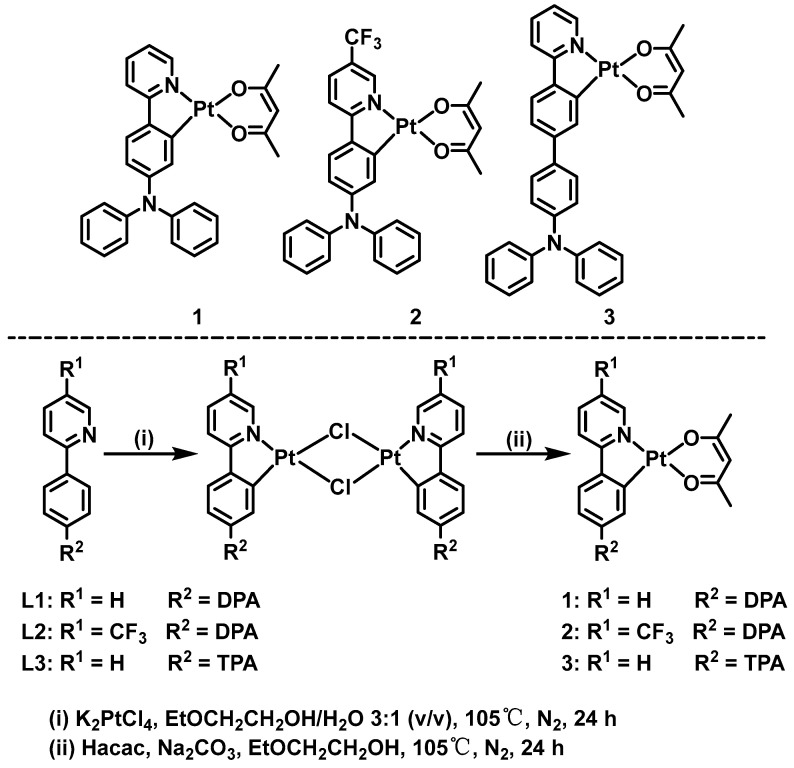
Structures and the synthetic routes of complexes **1**–**3.**

We are highly interested in disclosing the relevance between the structures and functions of cyclometalated Pt(II) and Ir(III) complexes [29,30,31,32,33,34]. Several cyclometalated Pt(II) complexes with AIPE properties were already developed for detecting PA in aqueous media [30,31]. However, further studies are needed on the development of the family of AIPE-active Pt(II) complexes to detect PA in aqueous media. Hence, three neutral Pt(II) complexes **1**–**3** were synthesized utilizing DPA-modified 2-phenylpyridine derivatives as the cyclometalating ligands. These Pt(II) complexes are AIPE-active due to their abundant intermolecular interactions and work well as luminescent probes to detect PA in THF/H_2_O. The structures of **1**–**3** are illustrated in Scheme 1.

## 2. Materials and Methods

### 2.1. Materials and Instruments

^1^H NMR spectra were obtained utilizing a Varian DLG400 (Palo Alto, CA, USA). Absorption spectra were recorded utilizing an Agilent Cary 100 UV-vis spectrophotometer (Santa Clara, CA, USA). Emission spectra were recorded utilizing a HITACHI F-7100 fluorescence spectrophotometer (Beijing, China). Phosphorescence decay traces were recorded utilizing an Edinburgh FLS1000 spectrometer (Livingston, Scotland, UK) in undeoxygenated THF/H_2_O. Scanning electron microscope (SEM) images were acquired utilizing a CIQTEK SEM5000 (Hefei, Anhui, China). Density functional theory (DFT) calculations were performed utilizing the B3LYP function. The LanL2DZ basis set was utilized for the platinum atom, and the 6–31G(d) basis set was applied for other atoms. All calculations were carried out utilizing the Gaussian 16 software package (New Haven, CT, USA).

### 2.2. Characterization of Complexes

The cyclometalating ligands and cyclometalated complexes were synthesized from the literature reported methodologies [9,35]. The synthetic routes of complexes **1**–**3** are shown in Scheme 1.

**1** [36]: Yield: 70%, a yellow solid. ^1^H NMR (400 MHz, CDCl_3_) *δ* 8.87 (d, *J* = 5.2 Hz, 1H), 7.71 (t, *J* = 7.7 Hz, 1H), 7.47 (d, *J* = 7.8 Hz, 1H), 7.29–7.24 (m, 5H), 7.24–7.18 (m, 5H), 7.05–6.97 (m, 3H), 6.76 (d, *J* = 8.4 Hz, 1H), 5.38 (s, 1H), 1.96 (s, 3H), 1.72 (s, 3H).

**2** [9]: Yield: 50%, a yellow solid. ^1^H NMR (400 MHz, CDCl_3_) *δ* 9.15 (s, 1H), 7.88 (dd, *J* = 8.6, 1.8 Hz, 1H), 7.50 (d, *J* = 8.7 Hz, 1H), 7.32–7.26 (m, 5H), 7.23–7.16 (m, 5H), 7.11–7.06 (m, 2H), 6.75 (dd, *J* = 8.5, 2.4 Hz, 1H), 5.40 (s, 1H), 1.99 (s, 3H), 1.72 (s, 3H). ^13^C NMR (100 MHz, CDCl_3_): *δ* 186.09, 184.10, 170.95, 149.82, 147.00, 144.39, 141.88, 135.37, 134.56, 129.24, 126.10, 125.39, 123.90, 121.65, 117.10, 116.49, 103.82, 102.40, 28.24, 26.71. HRMS (EI, *m*/*z*): calcd. for C_29_H_23_N_2_O_2_PtF_3_ [M]⁻: 683.1359, found: 683.1378.

**3** [37]: Yield: 62%, a yellow solid. ^1^H NMR (400 MHz, CDCl_3_) *δ* 9.00 (d, *J* = 6.1 Hz, 1H), 7.84–7.76 (m, 2H), 7.63–7.59 (m, 3H), 7.48 (d, *J* = 8.1 Hz, 1H), 7.34–7.26 (m, 5H), 7.15 (d, *J* = 7.5 Hz, 6H), 7.10 (t, *J* = 5.9 Hz, 1H), 7.03 (t, *J* = 7.3 Hz, 2H), 5.48 (s, 1H), 2.01 (d, *J* = 1.4 Hz, 6H).

### 2.3. Preparation of the Samples for Evaluating AIPE Properties and Detection of PA

The stock solutions for complexes **1**–**3** in THF (0.5 mM) were prepared, followed by the preparation of the suspensions of **1**–**3** (50 μM) in THF/H_2_O at different water fractions by addition of different volumes of THF and deionized water to the stock solution, and the emission spectra of **1**–**3** were subsequently measured. The suspensions of **1**–**3** (10 μM) in THF/H_2_O (**1**: *v*/*v* = 2:8, **2**: *v*/*v* = 1:9, **3**: *v*/*v* = 3:7) were prepared. Each time, the suspensions of complexes (10 μM, 2 mL) were added to the quartz cuvette. To obtain PA at various concentrations, the solutions were prepared in THF/H_2_O with concentrations in the range of 0.1 mM to 50 mM. Each time, 20 μL of PA solutions at different concentrations were added to the suspensions of **1**–**3** (10 μM, 2 mL), and their emission spectra were subsequently measured. The selectivity experiments to detect PA were conducted by the addition of solutions of analytes (30 mM), including 1,3-dinitrobenzene (1,3-DNB), *m*-cresol, 4-methoxyphenol (MEHQ), nitrobenzene (NB), nitromethane (NM), *p*-cresol, and phenol to the suspensions of **1**–**3**. In order to study the anti-interference ability for detecting PA, solutions of ionic compounds (30 mM, CH_3_COONa, KF, MgSO_4_, CuSO_4_, CaCl_2_, AlCl_3_, MnCl_2_, NiCl_2_, CoCO_3_, and KBr) were also prepared. For the competitive experiments and ion interference experiments, PA (30 mM, 20 μL) was added into the suspensions of **1**–**3,** which also contained the above analytes and ionic compounds. Several common water samples, including rainwater, tap water, and river water of Lingshui river from Dalian University of Technology, were chosen for detecting PA in real water samples. PA solutions (30 mM, 20 μL) were added to the suspensions of **1**–**3** in which common water samples were used instead of deionized water, and their emission spectra were subsequently measured.

**WARNING!** The nitroaromatic compounds used for optical measurement are highly explosive and should be handled safely and in small quantities.

## 3. Results and Discussion

### 3.1. AIPE Properties

**Figure 1:**
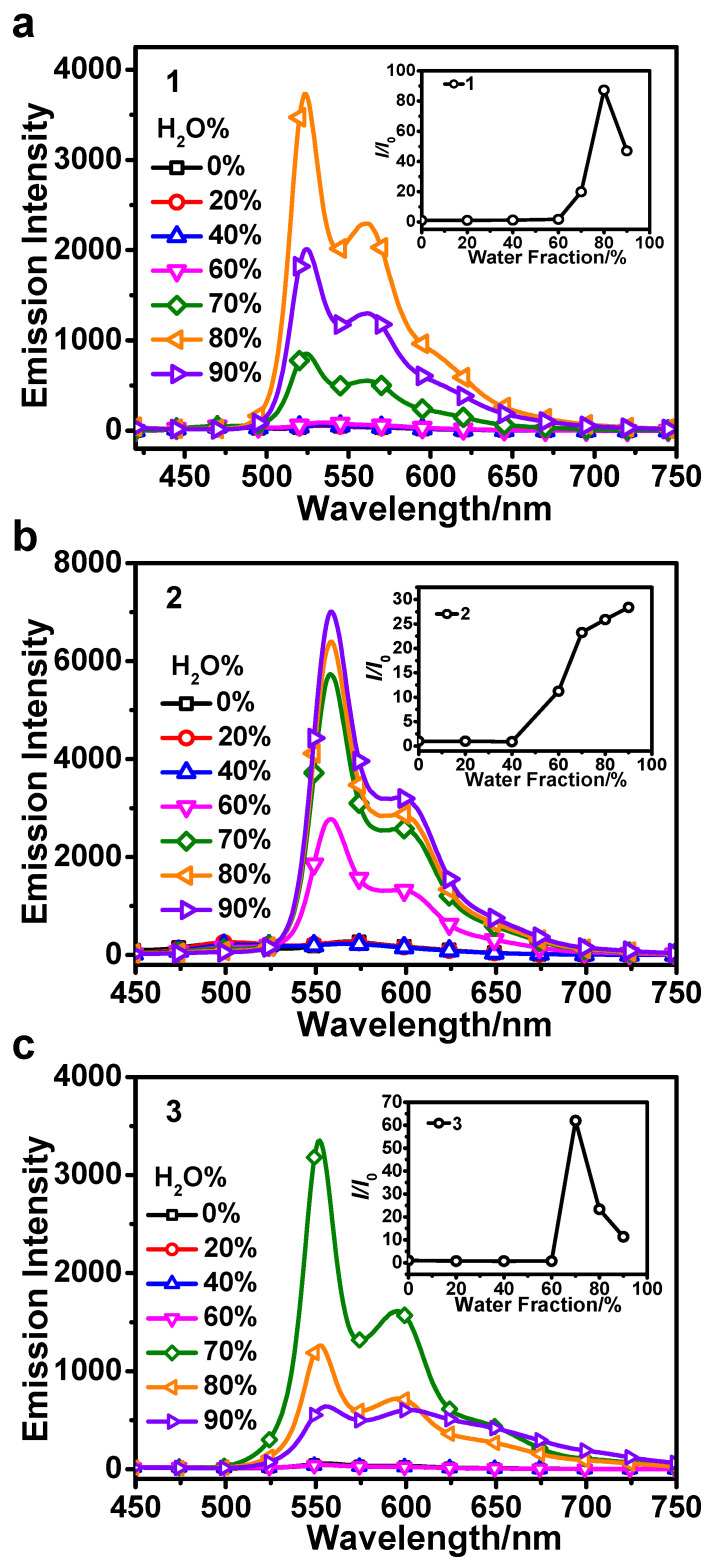
The emission spectra of **1** (**a**), **2** (**b**), and **3** (**c**) at 50 μmol/L in THF/H_2_O with different water fractions of 0–90%. Insert: The relationship between the relative intensities of **1**–**3** at the maxima emission wavelength and different water fractions. The excitation wavelength was 400 nm.

The fundamental photophysical properties of **1**–**3** have been studied at first and their details are presented in the Supporting Information (Figure S1 and Table S1). Subsequently, the emission spectra of **1**–**3** in THF/H_2_O at different water fractions were measured to explore their AIPE properties (Figure 1). The emission intensities of **1**–**3** are very weak below the water fraction of 60%. However, when the water fraction reaches 70–90%, the emission intensities are significantly enhanced. The maximum emission intensities of **1**–**3** are observed at water fractions of 80%, 90%, and 70%, which are approximately 82, 25, and 65 times greater than those in the THF solution, respectively. These results clearly indicate the presence of an evident AIPE phenomenon [38,39]. In THF solutions, the presence of freely rotatable benzene rings in the DPA group makes non-radiative transitions readily accessible. Meanwhile, the aggregation of **1**–**3** with increasing water fractions causes the motion of the DPA group to be restricted, which inhibits the non-radiative transitions and leads to their obvious AIPE activities [40,41,42].

### 3.2. Molecular Packing Modes

**Figure 2:**
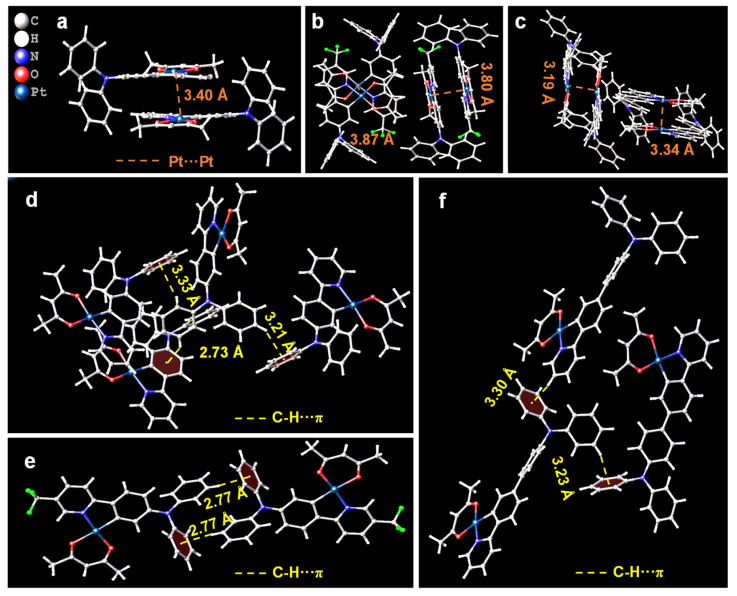
Intermolecular Pt···Pt interactions for **1** (**a**), **2** (**b**), and **3** (**c**). Intermolecular C-H···π hydrogen bonds for **1** (**d**), **2** (**e**), and **3** (**f**).

In order to elucidate the correlation of intermolecular interactions of **1**–**3** with their AIPE properties, a detailed analysis of the multiple intermolecular interactions for **1**–**3** was conducted by examining the molecular packing of their crystal structures. The crystal structures and corresponding data for **1–3** are illustrated in Figure S2 and Table S2. As shown in Figure 2a–c, the dimers of the complexes are formed by stacking in an anti-parallel mode of head-to-tail, demonstrating the characteristic of *J*-aggregate [18]. Notably, the mean Pt···Pt distances of **1**–**3** are 3.40, 3.84, and 3.27 Å, respectively, indicating substantial Pt···Pt interactions within the crystal structures, which facilitate the orderly stacking of molecules. Meanwhile, the presence of abundant intermolecular C-H···π hydrogen bonds for **1**–**3** effectively restricts the rotation of benzene rings in the DPA group (Figure 2d–f). Furthermore, the crystal stacks for **2** and **3** reveal other intermolecular interactions with C-H···O hydrogen bonds both in **2** and **3** and C-H···F hydrogen bonds also in **2** (Figures S3 and S4, Supporting Information). These intermolecular interactions allow the molecules to aggregate tightly, effectively restricting the intramolecular motion of **1**–**3**, suppressing the occurrence of non-radiative pathways, and leading to prominent AIPE properties.

### 3.3. Theoretical Calculations

**Figure 3:**
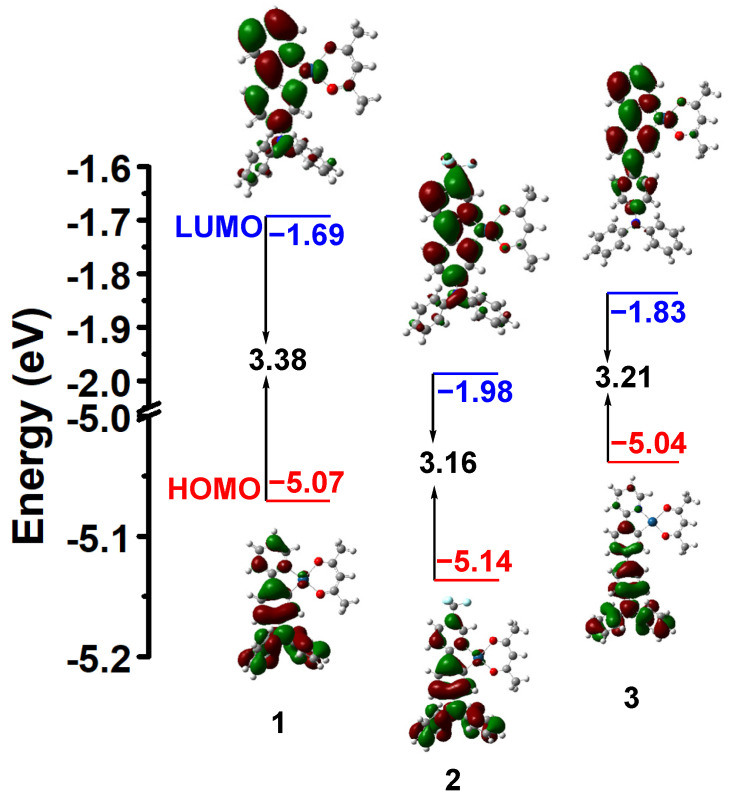
Calculated energy-level diagram and contour plots of the HOMO and LUMO for **1**–**3**.

As seen in Figure 3, the molecular orbital topologies of **1**–**3** are similar. The LUMOs of **1**–**3** are mainly located on 2-phenylpyridine of the cyclometalating ligands and the Pt center. The HOMOs of **1** and **2** are predominantly located on the cyclometalating ligands and Pt center, while the HOMO of **3** is predominantly distributed on the triphenylamino group of the cyclometalating ligand. Furthermore, the energy gaps for **1–3** are calculated to be 3.38, 3.16, and 3.21 eV, respectively. The energy gaps of **2** and **3** reduce compared to that of **1**, which is consistent with the experimental results.

### 3.4. Sensing of PA

**Figure 4:**
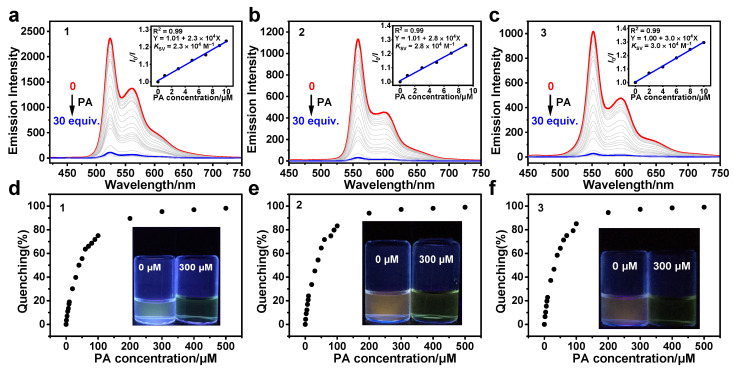
The emission spectra of **1** (**a**), **2** (**b**), and **3** (**c**) in THF/H_2_O (**1**: *v*/*v* = 2:8, **2**: *v*/*v* = 1:9, **3**: *v*/*v* = 3:7, 10 μM) with PA at different concentrations. Insert: The SV plots of **1**–**3** at the PA concentration range of 0–10 μM. Quenching percentages of **1** (**d**), **2** (**e**), and **3** (**f**) after adding PA at various concentrations. Insert: The photos of **1**–**3** at PA concentrations of 0 and 300 μM under 365 nm UV light. The excitation wavelength was 400 nm.

Complexes **1**–**3** exhibit obvious AIPE phenomenon and good photostabilities, which suggests that they could potentially serve as probes to detect PA in aqueous media (Figure 1 and Figure S5). Thus, their emission quenching properties were investigated by adding PA solutions at different concentrations to the suspensions of **1**–**3** in THF/H_2_O (**1**: *v*/*v* = 2:8, **2**: *v*/*v* = 1:9, **3**: *v*/*v* = 3:7). As shown in Figure 4a–c, the emission intensities of **1**–**3** consistently decline with increasing PA concentration. When the PA concentration is 10 μM (1 equiv.), the quenching efficiencies of **1**–**3** are measured to be 36.2%, 28.0%, and 46.4%. As the PA concentration increases to 300 μM (30 equiv.), the emissions of **1**–**3** are nearly negligible, with quenching efficiencies above 95% (Figure 4d–f).

The quenching effects of **1**–**3** for PA were investigated by analyzing the Stern–Volmer (SV) plots of *I*_0_/*I* (*I*_0_ and *I* represent the maximum emission intensities without and with PA, respectively) vs. PA concentration [43,44]. The SV plots of **1**–**3** exhibit a strong linearity within the PA concentration ranging from 0–10 μM (Figure 4a–c, inset), whereas a nonlinear relationship is shown within the PA concentration ranging from 0–500 μM (Figure S6, Supporting Information). In the PA concentration ranging from 0–10 μM, the emission quenching and PA concentration were quantitatively analyzed using the SV equation: *I*_0_/*I* = *K*_SV_[Q] + 1 [45]. The values of *K*_SV_ for **1**–**3** were measured to be 2.3 × 10^4^, 2.8 × 10^4^, and 3.0 × 10^4^ M^−1^, respectively. In addition, the limit of detection (LOD) can be calculated utilizing the equation: LOD = 3*σ*/*K* [46]. Based on Table S3 and Figure S7, the LODs for **1**–**3** were determined to be 70, 100, and 90 nM, respectively. In contrast to most of the previously reported Pt(II) complexes for the detection of PA in organic solvents, **1**–**3** exhibit simpler molecular structures and lower limits of detection for PA in aqueous media (Table S4). These results suggest that **1**–**3** can be efficiently deployed to detect PA in aqueous media.

**Figure 5:**
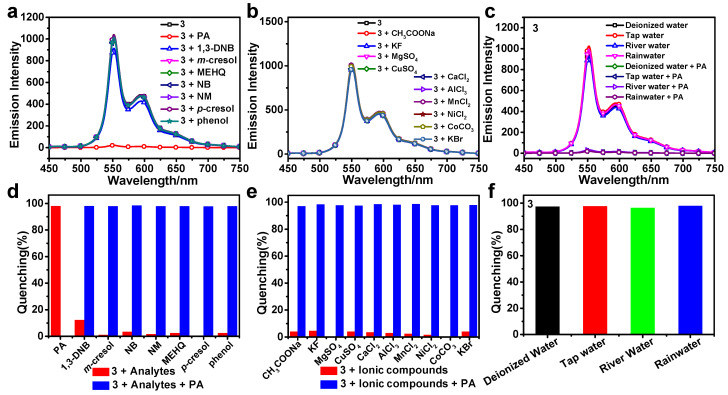
The emission spectra of **3** in THF/H_2_O (*v*/*v* = 3:7, 10 μM) with different analytes (**a**), ionic compounds (**b**), and common water samples (**c**) present. Quenching percentages of **3** with different analytes (**d**) and ionic compounds (**e**) before (red) and after (blue) addition of PA. (**f**) Quenching percentage of **3** towards PA in common water samples. The excitation wavelength was 400 nm.

The selective and competitive experiments, ion interference experiments, and experiments with different water samples were conducted to explore the practical applicability of the sensors (Figure 5, Figures S8 and S9).

Firstly, various analytes (1,3-DNB, *m*-cresol, MEHQ, NB, NM, *p*-cresol, and phenol) were chosen for selective and competitive experiments. After the addition of other analytes, **1**–**3** exhibit similar emission spectra (Figure 5a, Figures S8a and S9a). In addition, the quenching percentages of **1**–**3** in the presence of different analytes are much lower than that of PA (Figure 5d, Figures S8d and S9d). These results show that these complexes can effectively identify PA among the above analytes, demonstrating good selectivity for PA. The luminescence quenching of **1**–**3** in the presence of PA may be attributed to the introduction of the electron-rich DPA group that makes **1**–**3** good electron donors, whereas PA serves as an electron acceptor, promoting the photo-induced electron transfer (PET) process [31,47]. Subsequently, competitive experiments were carried out by the addition of PA solutions at 30 equiv. to the suspensions of **1**–**3** with other analytes present. Figure 5d, Figures S8d and S9d indicate that PA still leads to the luminescence quenching of **1**–**3** in the presence of other analytes, which proves that complexes are unaffected by the above analytes in detecting PA and exhibit excellent anti-interference ability.

Next, ion interference experiments were conducted using CH_3_COONa, KF, MgSO_4_, CuSO_4_, CaCl_2_, AlCl_3_, MnCl_2_, NiCl_2_, CoCO_3_, and KBr as ionic compounds. The emission intensities of **1**–**3** remain almost unchanged after the addition of different ionic compound solutions, indicating that the presence of ions does not affect the luminescent properties of the complexes (Figure 5b, Figures S8b and S9b). However, when PA solutions at 30 equiv. are added to the suspensions of **1**–**3** with different ionic compounds, the quenching efficiencies of the complexes significantly increase, indicating that PA can quench the luminescence of the complexes in the presence of ionic compounds (Figure 5e, Figures S8e and S9e).

Lastly, the detection performances of **1**–**3** in THF/H_2_O using common water samples (tap water, rainwater, and river water) instead of deionized water were tested. The emission spectra of **1**–**3** do not show a significant change in common water samples in comparison to those in deionized water (Figure 5c, Figures S8c and S9c). The quenching percentages suggest that **1**–**3** perform satisfactorily in common water samples (Figure 5f, Figures S8f and S9f).

### 3.5. Sensing Mechanism

**Figure 6:**
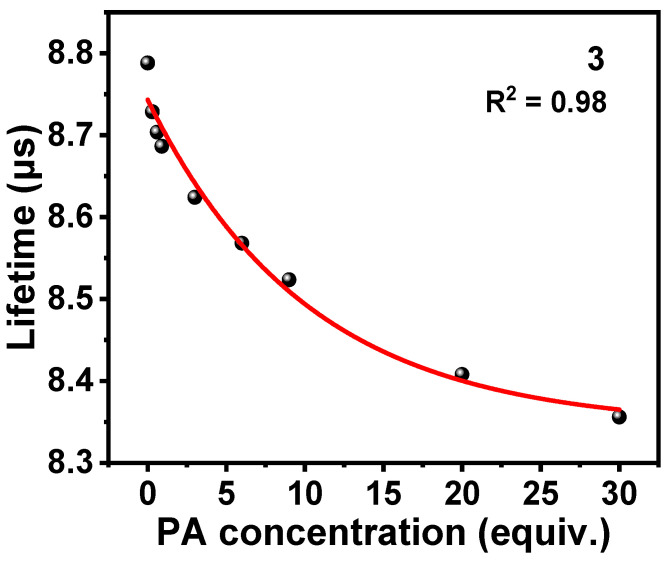
Changes in phosphorescence lifetime of **3** in THF/H_2_O (*v*/*v* = 3:7, 10 μM) after adding PA at different concentrations.

The static and dynamic quenching processes are usually distinguished by whether the lifetime of the luminescent probe changes without and with the quencher [47]. Therefore, the phosphorescence decay traces of **3** after the addition of PA at different concentrations were measured to investigate the quenching mechanism of **3** for PA, as shown in Figure S10. Subsequently, the phosphorescence decay traces were fitted to obtain the lifetimes of **3** with PA at different concentrations present. As shown in Figure 6, the lifetime of **3** is decreasing continuously with the increase of PA concentration. With PA concentration up to 30 equiv., the lifetime of **3** decreases from the initial 8.79 μs to 8.36 μs, which successfully proves the existence of dynamic quenching in the luminescence quenching process of complex **3**.

**Figure 7:**
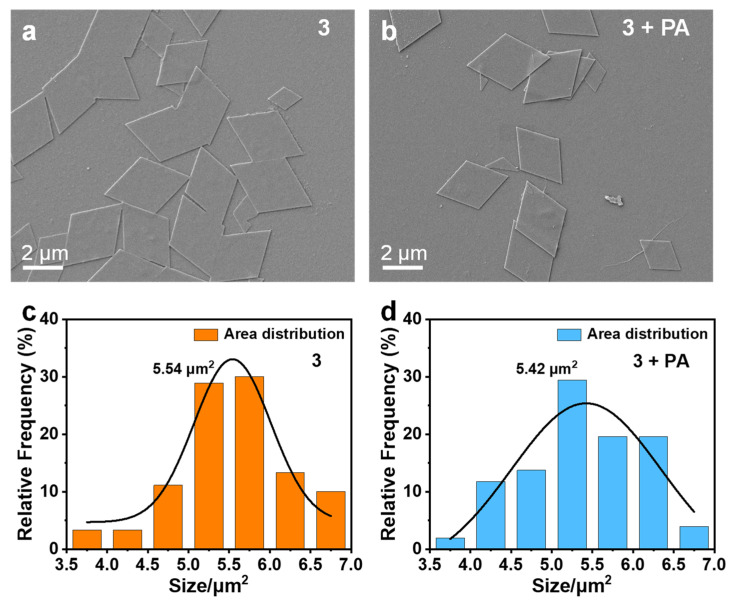
SEM images and area distribution of the aggregates of **3** in THF/H_2_O (*v*/*v* = 3:7, 10 μM) before (**a**,**c**) and after (**b**,**d**) the addition of PA (30 equiv.).

Firstly, the HRMS and NMR spectra of **1**–**3** demonstrate that their structures are correct (Figures S11–S15). Then, scanning electron microscope (SEM) experiments were utilized to verify the formation of the aggregates and to probe their interaction with PA. Figure 7a,c show that **3** forms regular sheet-like aggregates in THF/H_2_O (*v*/*v* = 3:7, 10 μM), and their average area is 5.54 μm^2^. The morphology and area distribution of the aggregates for **3** obtained after the addition of PA (30 equiv.) are shown in Figure 7b,d, and they still remain in the same form of regular sheets with an average area of 5.42 μm^2^. The addition of PA has no significant influence on the morphologies and sizes of the aggregates of **3**, indicating that there is no strong interaction between **3** and PA to form a ground state complex.

**Figure 8:**
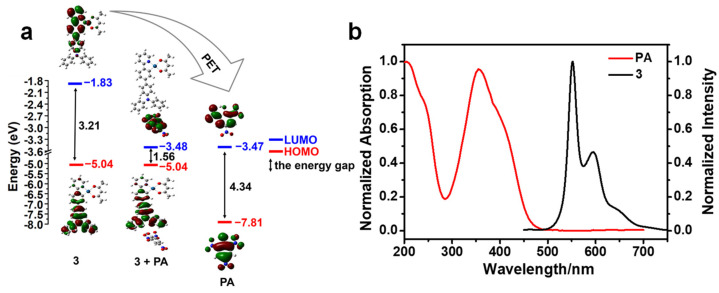
(**a**) Calculated energy level diagram of **3**, PA, and adduct (**3** + PA). (**b**) the normalized absorption spectrum of PA and normalized emission spectrum of **3**. The excitation wavelength was 400 nm.

Based on the PET process, if the LUMO energy of PA is lower than those of sensors, the excited state electrons of sensors can be transferred to the LUMO of PA. The sensors’ luminescence is reduced as the electrons on the LUMO of PA gradually return to their ground state through a non-radiative pathway. Figure 8a clearly demonstrates that the LUMO energy of **3** is obviously higher than that of PA, indicating the occurrence of the PET process [48,49]. The adduct (**3** + PA) exhibits favorable stability due to its lowest energy gap. The emission spectrum of **3** does not overlap with the absorption spectrum of PA, suggesting that there is no Förster Resonance Energy Transfer during the detection process (Figure 8b). The results indicate that PET has a major contribution to the phosphorescence quenching for complexes.

## 4. Conclusions

In conclusion, three AIPE-active cyclometalated complexes **1**–**3** with a DPA group were successfully synthesized. The crystal structures of these complexes indicate that the intermolecular Pt···Pt interactions and hydrogen bonds are the main contributions to their AIPE properties. In addition, **1**–**3** exhibit high selectivity and sensitivity for detecting PA in aqueous media at quenching constants of 2.3 × 10^4^, 2.8 × 10^4^, and 3.0 × 10^4^ M^−1^, and LODs of 70, 100, and 90 nM, respectively. In comparison to most of the previously reported Pt(II) complexes for detecting PA in organic solvents, **1**–**3** exhibit simpler molecular structures and lower limits of detection for PA in aqueous media. These complexes possess excellent ability for detecting PA in complicated environments and also show promising applicability in common water samples. The mechanism of the quenching of luminescence is PET. These results provide useful insights into the design of novel Pt(II) complexes to detect PA for practical application.

## Data Availability

Data are contained within the article.

## Supplementary Materials

The following supporting information can be downloaded at: https://www.mdpi.com/article/10.3390/ma17174366/s1, Figure S1: (**a**) Normalized absorption (solid trace) and emission (dash trace) spectra of **1**–**3** at room temperature (*c* = 50 μM in THF). (**b**) Normalized emission spectra of **1**–**3** in the solid state. The excitation wavelength was 400 nm. Table S1: Photophysical data of **1**–**3**. Figure S2: Crystal structures of **1** (**a**), **2** (**b**), and **3** (**c**). Figure S3: (**a**) Intermolecular C-H···O hydrogen bonds and (**b**) intermolecular C-H···F hydrogen bonds of **2**. Figure S4: Intermolecular C-H···O hydrogen bonds of **3**. Table S2: Crystal data for complexes **1**–**3**. Figure S5: The emission spectra of **1** (**a**), **2** (**b**), and **3** (**c**) in 11 blank samples. The excitation wavelength was 400 nm. Table S3: The emission intensities of **1** at 523 nm, **2** at 558 nm, and **3** at 552 nm in 11 blank samples. Figure S6: The Stern–Volmer plots of **1** (**a**), **2** (**b**), and **3** (**c**) for PA. Figure S7: The linear graphs of the emission intensities of **1** (**a**), **2** (**b**), and **3** (**c**) vs. the concentration of PA. Table S4: Some reported sensors for the detection of PA. Figure S8: The emission spectra of **1** in THF/H_2_O (*v*/*v* = 2:8, 10 μM) with different analytes (**a**), ionic compounds (**b**), and common water samples (**c**) present. Quenching percentages of **1** with different analytes (**d**), ionic compounds (**e**) before (red) and after (blue) the addition of PA. (**f**) Quenching percentage of **1** towards PA in common water samples. The excitation wavelength was 400 nm. Figure S9: The emission spectra of **2** in THF/H_2_O (*v*/*v* = 1:9, 10 μM) with different analytes (**a**), ionic compounds (**b**), and common water samples (**c**) present. Quenching percentages of **2** with different analytes (**d**), ionic compounds (**e**) before (red) and after (blue) the addition of PA. (**f**) Quenching percentage of **2** towards PA in common water samples. The excitation wavelength was 400 nm. Figure S10: Phosphorescence decay traces of **3** in THF/H_2_O (*v*/*v* = 3:7, 10 μM) after addition of PA at different concentrations. Figure S11: The ^1^H NMR spectrum of **1** in CDCl_3_. Figure S12: The ^1^H NMR spectrum of **2** in CDCl_3_. Figure S13: The ^13^C NMR spectrum of **2** in CDCl_3_. Figure S14: The HRMS of **2**. Figure S15: The ^1^H NMR spectrum of **3** in CDCl_3_.
