# Supplementary material for: Diphenylamino-Modified Neutral Pt(II) Complexes: Their Aggregation-Induced Phosphorescent Emission and Picric Acid-Sensing Properties
Source: Materials (Basel). 2024 Sep 3;17(17):4366. doi: 10.3390/ma17174366 (PMC11395801; doi:10.3390/ma17174366)
Supplement: Supplementary file 1 [file materials-17-04366-s001.zip › materials-3174728-supplementary.pdf]

materials

MDPI

Supplementary data for

Diphenylamino-Modified Neutral Pt(II) Complexes: Their Aggregation-Induced Phosphorescent Emission and Picric Acid-Sensing Properties

Qinglong Zhang [1], Yingying Yan [1], Rui Cai [1], Xiao-Na Li [2,*] and Chun Liu [1,*]

[1] State Key Laboratory of Fine Chemicals, Frontier Science Center for Smart Materials, School of Chemical Engineering, Dalian University of Technology, Linggong Road 2, Dalian 116024, China; zhangqinglong@mail.dlut.edu.cn (Q.Z.); yanyingying@mail.dlut.edu.cn (Y.Y.); cairui@dlut.edu.cn (R.C.)

[2] School of Environmental Science and Technology, Key Laboratory of Industrial Ecology and Environmental Engineering (MOE), Dalian University of Technology, Dalian 116024, China

* Correspondence: klieee@dlut.edu.cn (X.-N.L.); cliu@dlut.edu.cn (C.L.)

Citation: Zhang, Q.; Yan, Y.; Cai, R.; Li, X.; Liu, C. Diphenylamino-Modified Neutral Pt(II) Complexes: Their Aggregation-Induced Phosphorescent Emission and Picric Acid-Sensing Properties. *Materials* **2024**, *17*, 4366. https://doi.org/10.3390/ma17174366

Academic Editor: F. Rivera-López

Received: 9 August 2024

Revised: 28 August 2024

Accepted: 30 August 2024

Published: 3 September 2024

Copyright: © 2024 by the authors. Licensee MDPI, Basel, Switzerland. This article is an open access article distributed under the terms and conditions of the Creative Commons Attribution (CC BY) license (https://creativecommons.org/licenses/by/4.0/).

Contents:

Photophysical properties *Pages S1-S2.*

Molecular packing modes of complexes **1–3** *Pages S2-S3.*

PA detection of complexes **1–3** *Pages S4-S6.*

Phosphorescence decay traces of **3** in detecting PA *Pages S7.*

NMR spectra and HRMS of complexes **1–3** *Pages S7-S9.*

References *Page S9-S10.*

Photophysical properties

The UV-vis absorption spectra of **1–3** in THF at room temperature are presented in Figure S1a, and the detailed photophysical data are listed in Table S1. All complexes exhibit the strong absorption bands below 300 nm, which are assigned to a typical ligand-centered ^{1}LC ($^1\pi$-π^*) transition. The lower energy absorption bands at about 350–450 nm are derived from mixing among metal-to-ligand ^{1}MLCT/^{3}MLCT and ligand-to-ligand charge transfer ^{1}LLCT/^{3}LLCT together with ligand-centered ^{3}LC ($^3\pi$-π^*) transitions, which are similar to the previously reported literature [1].

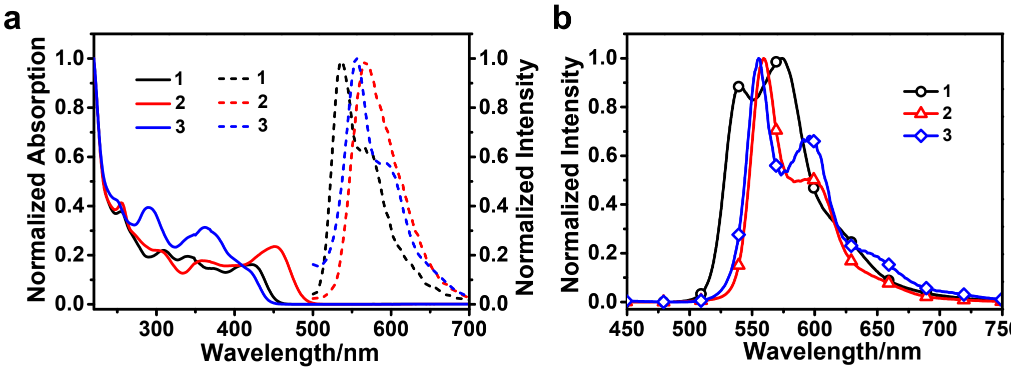

Figure S1. (a) Normalized absorption (solid trace) and emission (dash trace) spectra of **1–3** at room temperature (c = 50 μM in THF). (b) Normalized emission spectra of **1–3** in the solid state. The excitation wavelength was 400 nm.

Figure S1a presents the emission spectra of **1–3** in THF. Efficient phosphorescence emission peaks of 536, 567, and 556 nm are observed for **1–3**. In comparison to **1**, the maximum emission wavelengths of **2** and **3** are red-shifted due to the presence of trifluoromethyl and triphenylamine group. In the solid state, the emission maxima of **1–3**

are determined as 573, 559, and 555 nm, respectively (Figure S1b). The phosphorescence quantum yields of **1–3** are 11%, 14%, and 4%, respectively. The results display that the structures of cyclometalating ligands have a substantial impact on the photophysical properties of the complexes.

Table S1. Photophysical data of **1–3**.

Complexes	λ_{abs} [a] (nm)	λ_{em} [b] (nm)	λ_{em} [c] (nm)	Φ_{PL} [d]
**1**	254(1.42), 309(0.82), 340(0.72), 422(0.59)	**536**, 568	540, **573**	11%
**2**	256(1.63), 306(0.84), 357(0.68), 451(0.92)	**567**	**559**, 600	14%
**3**	250(1.76), 290(1.64), 361(1.29), 420(0.50)	**556**, 596	**555**, 595	4%

[a] Measured in THF at a concentration of 50 μM and extinction coefficients (10^4 M^{-1} cm^{-1}) are shown in parentheses. [b] The emission maxima of the complexes in THF. [c] The emission maxima of the complexes in the solid state. [d] The quantum yields (Φ_{PL}) in deoxygenated CH_2Cl_2 were measured with [Ir(ppy)$_2$(acac)] (Φ_{PL} = 0.34) as a standard. All results were measured at ambient temperature.

Molecular packing modes of complexes 1–3

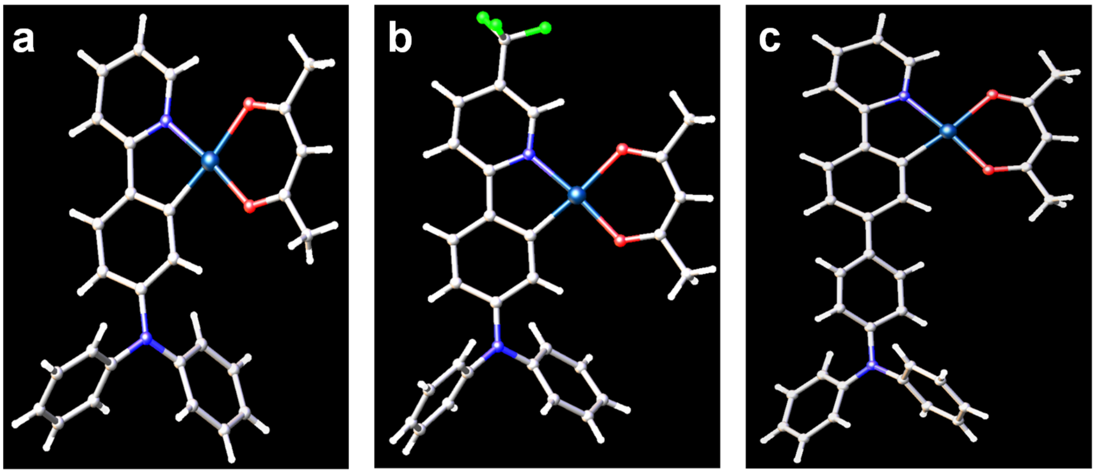

Figure S2. Crystal structures of **1** (a), **2** (b), and **3** (c).

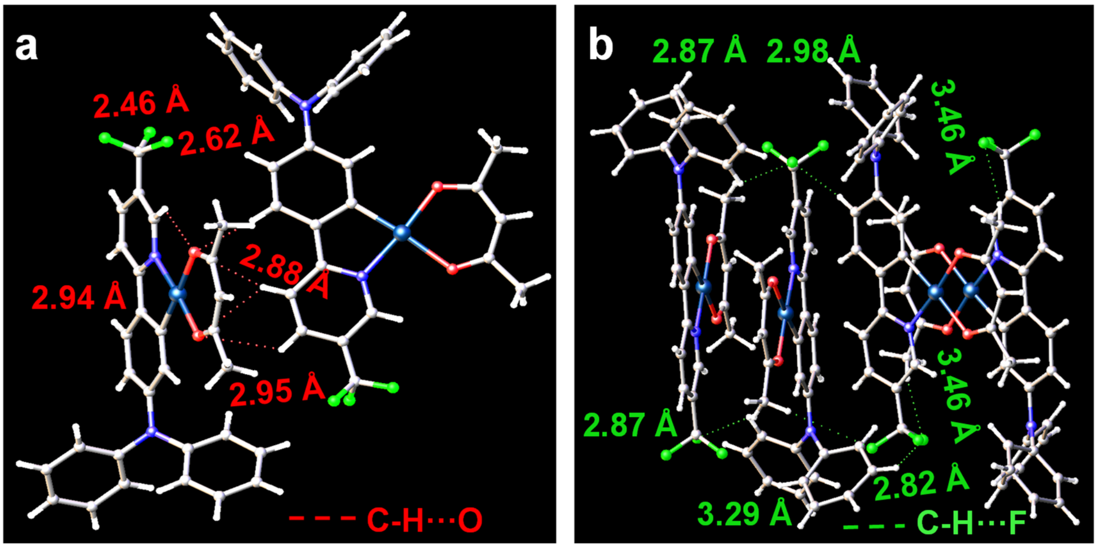

Figure S3. (a) Intermolecular C-H···O hydrogen bonds and (b) intermolecular C-H···F hydrogen bonds of **2**.

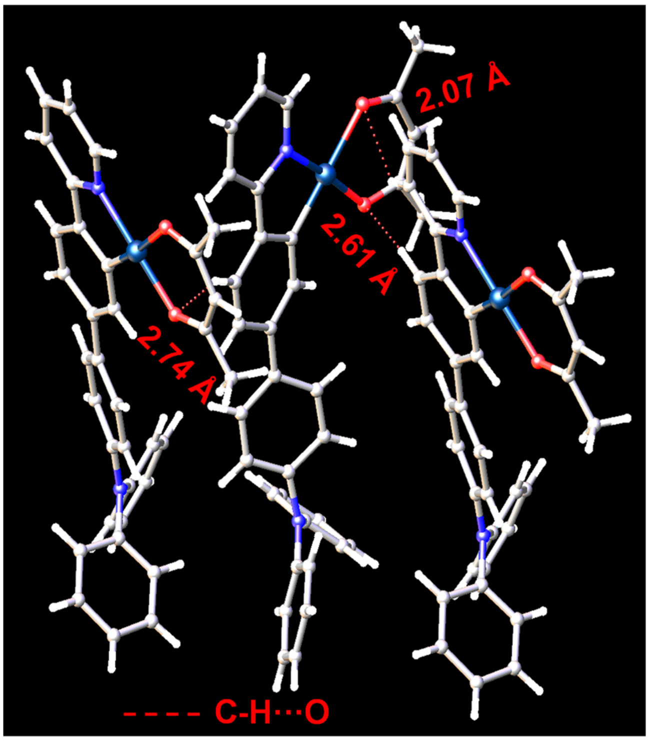

Figure S4. Intermolecular C-H···O hydrogen bonds of **3**.

Table S2. Crystal data for complexes **1**–**3**.

**Complexes**	**1**	**2**	**3**
**Formula**	$C_{28}H_{24}O_2N_2Pt$	$C_{29}H_{23}O_2N_2F_3Pt$	$C_{34}H_{28}O_2N_2Pt$
**Crystal System**	Monoclinic	Triclinic	Triclinic
**Temperature**	296 K	296 K	293 K
**Space group**	P2(1)/c	P-1	P-1
**Cell lengths (Å)**	a = 13.446(4)<br>b = 9.606(3)<br>c = 18.330(6)	a = 12.1092(4)<br>b = 12.7133(4)<br>c = 18.3191(6)	a = 10.4098(14)<br>b = 15.594(2)<br>c = 16.792(2)
**Cell angles (°)**	α = 90<br>β = 103.229(4)<br>γ = 90	α = 78.388(3)<br>β = 72.068(2)<br>γ = 89.806(2)	α= 90.110(2)<br>β = 93.084(2)<br>γ = 91.575(2)
**Cell volume (Å^3)**	2304.6(13)	2623.08(15)	2720.8(6)
**Z**	4	2	2
**Density (g/cm^3)**	1.774	1.731	1.689
**F (000)**	1200.0	1328.0	1360.0
**hmax, kmax, lmax**	17, 12, 23	14, 15, 21	12, 15, 18
**Absorption coefficient (mm^{-1})**	6.117	5.399	5.192
**R (int)**	0.0255	0.0581	0.0831
**Data/restraints/ parameters**	5144/0/300	9219/54/671	9443/48/703
**Goodness-of-fit on F^2**	1.049	1.030	0.921
R_1[a] $[I > 2\sigma(I)]$	0.0224	0.0478	0.0729
wR_2[b] $[I > 2\sigma(I)]$	0.0502	0.0725	0.1119
R_1[a] **(all data)**	0.0313	0.0945	0.1628
wR_2[b] **(all data)**	0.0532	0.0806	0.0930
**CCDC number**	2071354	918648	762460

$$^{[a]}R_1 = \sum \| F_o | - | F_c \| / \sum | F_o |$$

$$^{[b]}wR_2 = [\sum w(F_o^2 - F_c^2)^2 / \sum w(F_o^2)^2]^{1/2}$$

PA detection of complexes 1–3

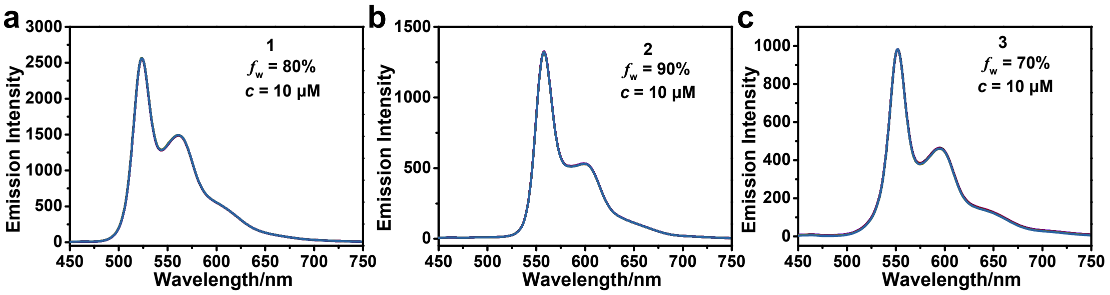

Figure S5. The emission spectra of **1** (a), **2** (b), and **3** (c) in 11 blank samples. The excitation wavelength was 400 nm.

Table S3. The emission intensities of **1** at 523 nm, **2** at 558 nm, and **3** at 552 nm in 11 blank samples.

Complexes	1	2	3
X_1	2560.8	1316.8	979.5
X_2	2560.4	1316.9	979.8
X_3	2560.2	1316.7	980.0
X_4	2562.1	1317.9	980.3
X_5	2560.5	1318.4	981.0
X_6	2561.9	1318.9	981.3
X_7	2559.8	1319.4	981.7
X_8	2559.8	1317.7	980.4
X_9	2558.6	1317.4	980.8
X_{10}	2559.6	1317.5	981.2
X_{11}	2560.6	1316.7	981.5
X	2560.4	1317.7	980.8

The values of σ for **1–3** were calculated according to the following equation:

$\sigma = [\sum(X_i-X)^2/(n-1)]^{0.5}$

X_i (i = 1, 2, 3…11) represents the emission intensity of each blank sample, X represents the mean value of the emission intensity, n represents the number of blank samples.

According to the above formula, the values of σ for **1–3** were calculated to be 0.9529, 0.8752, and 0.7041.

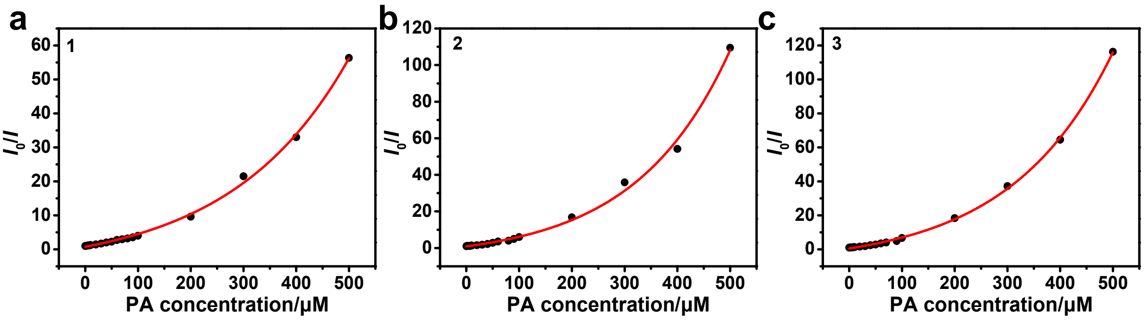

Figure S6. The Stern–Volmer plots of **1** (a), **2** (b), and **3** (c) for PA.

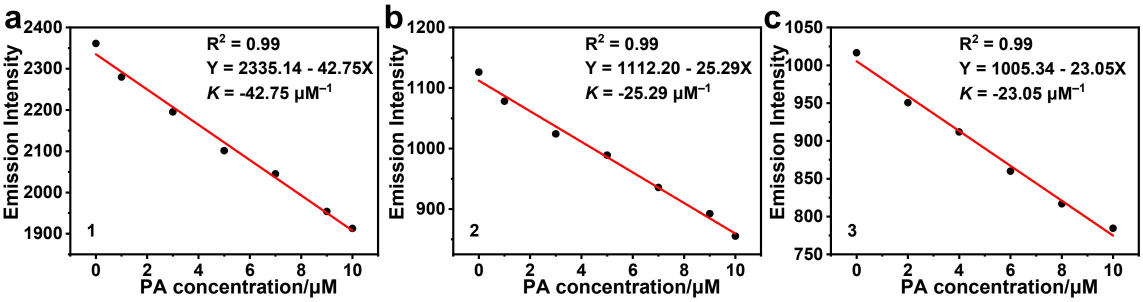

Figure S7. The linear graphs of the emission intensities of **1** (a), **2** (b), and **3** (c) vs. the concentration of PA.

Table S4. Some reported sensors for the detection of PA.

Probes	Solvent	LOD	K_{SV}/M^{-1}	References
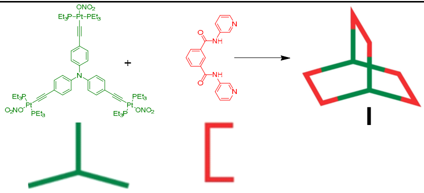	CH_2Cl_2/DMF (v/v, 4:1)	-	4.8×10^4	[2]
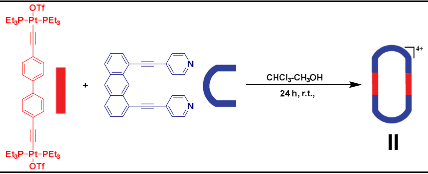	$CHCl_3/CH_3OH$ (v/v, 1:1)	-	5.0×10^6	[3]
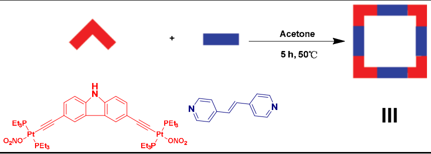	DMF	-	5.0×10^4	[4]
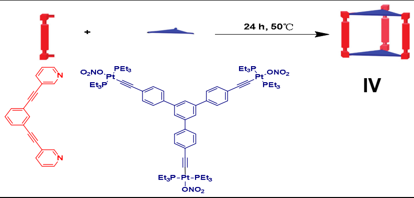	$CHCl_3$	-	3.6×10^4	[5]
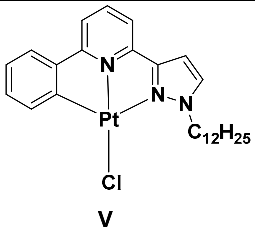	Micellar media	1.0 μM	1.1×10^4	[6]
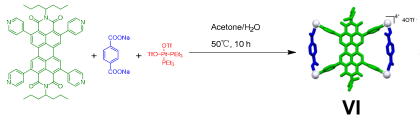	CH_3CN	2.3 μM	6.8×10^4	[7]
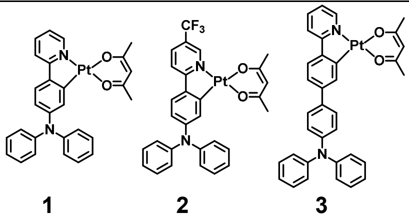	THF/H_2O **1**: (v/v, 2:8) **2**: (v/v, 1:9) **3**: (v/v, 3:7)	**1**: 70 nM **2**: 100 nM **3**: 90 nM	**1**: 2.3×10^4 **2**: 2.8×10^4 **3**: 3.0×10^4	**This work**

- no values reported.

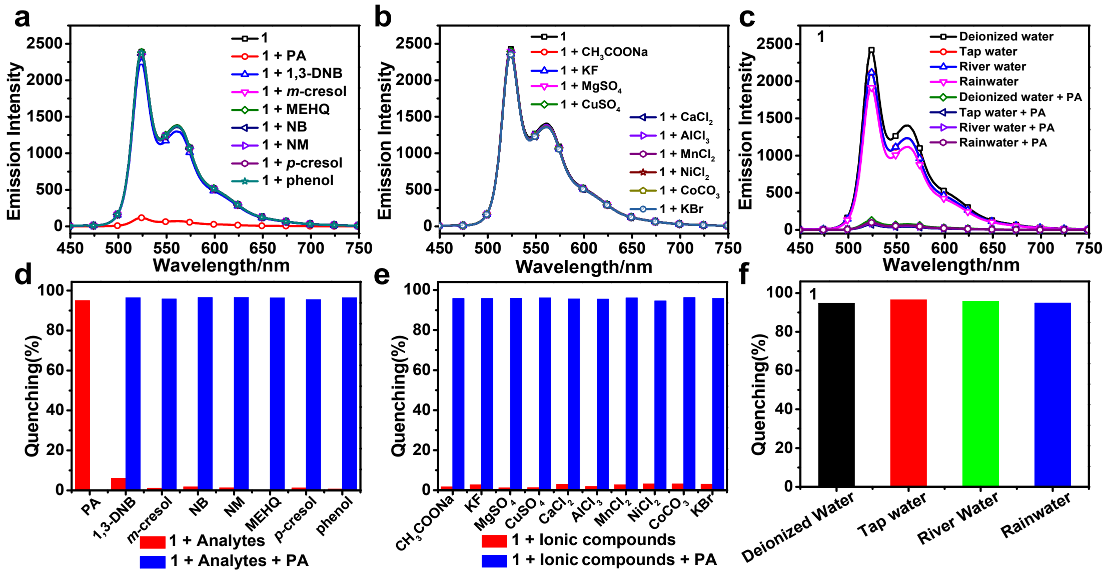

Figure S8. The emission spectra of **1** in THF/H_2O (v/v = 2:8, 10 μM) with different analytes (a), ionic compounds (b), and common water samples (c) present. Quenching percentages of **1** with different analytes (d), ionic compounds (e) before (red) and after (blue) the addition of PA. (f) Quenching percentage of **1** towards PA in common water samples. The excitation wavelength was 400 nm.

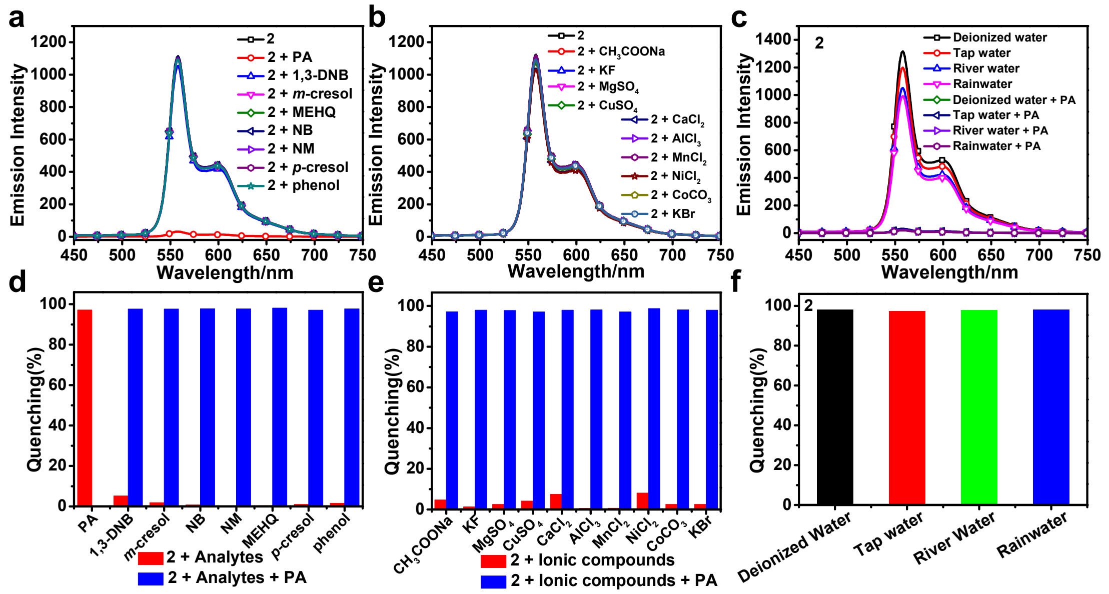

Figure S9. The emission spectra of **2** in THF/H_2O (v/v = 1:9, 10 μM) with different analytes (a), ionic compounds (b), and common water samples (c) present. Quenching percentages of **2** with different analytes (d), ionic compounds (e) before (red) and after (blue) the addition of PA. (f) Quenching percentage of **2** towards PA in common water samples. The excitation wavelength was 400 nm.

Phosphorescence decay traces of 3 in detecting PA

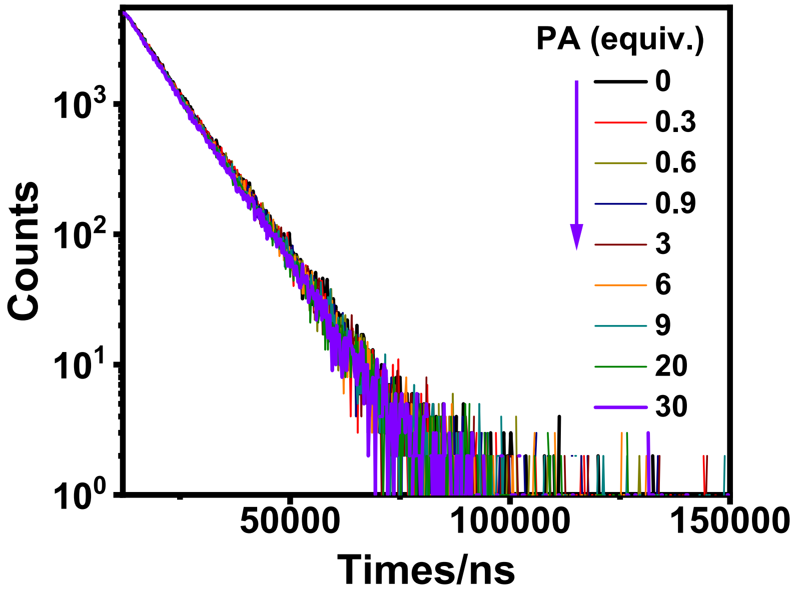

Figure S10. Phosphorescence decay traces of **3** in THF/H_2O (v/v = 3:7, 10 μM) after addition of PA at different concentrations.

NMR spectra and HRMS of complexes 1–3

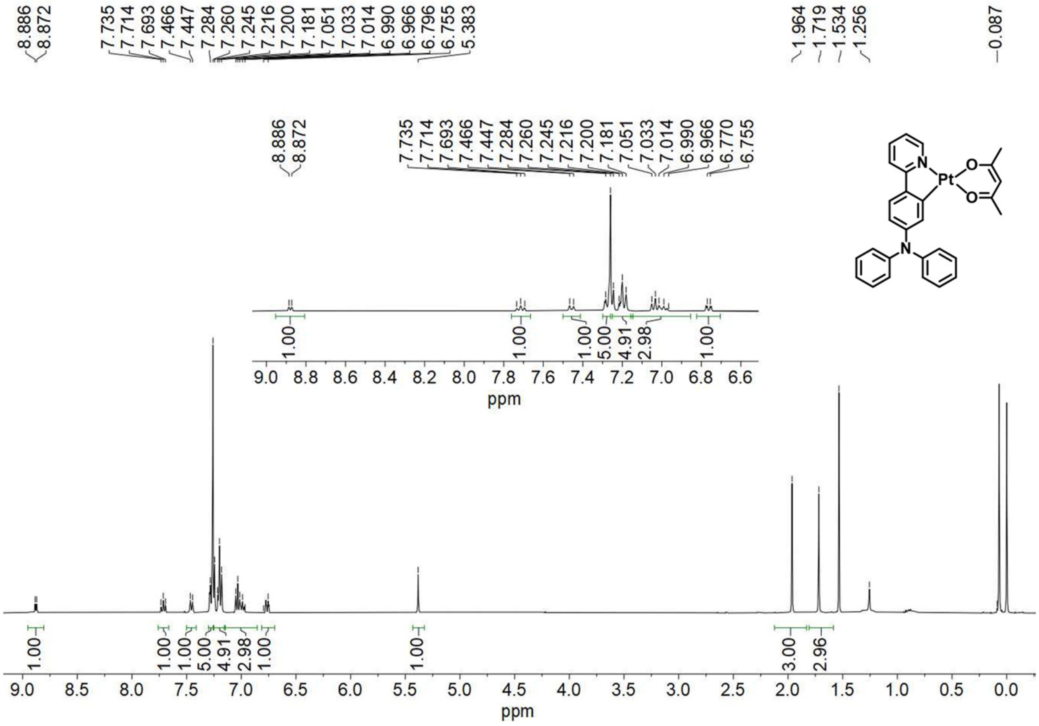

Figure S11. The ^{1}H NMR spectrum of **1** in $CDCl_3$.

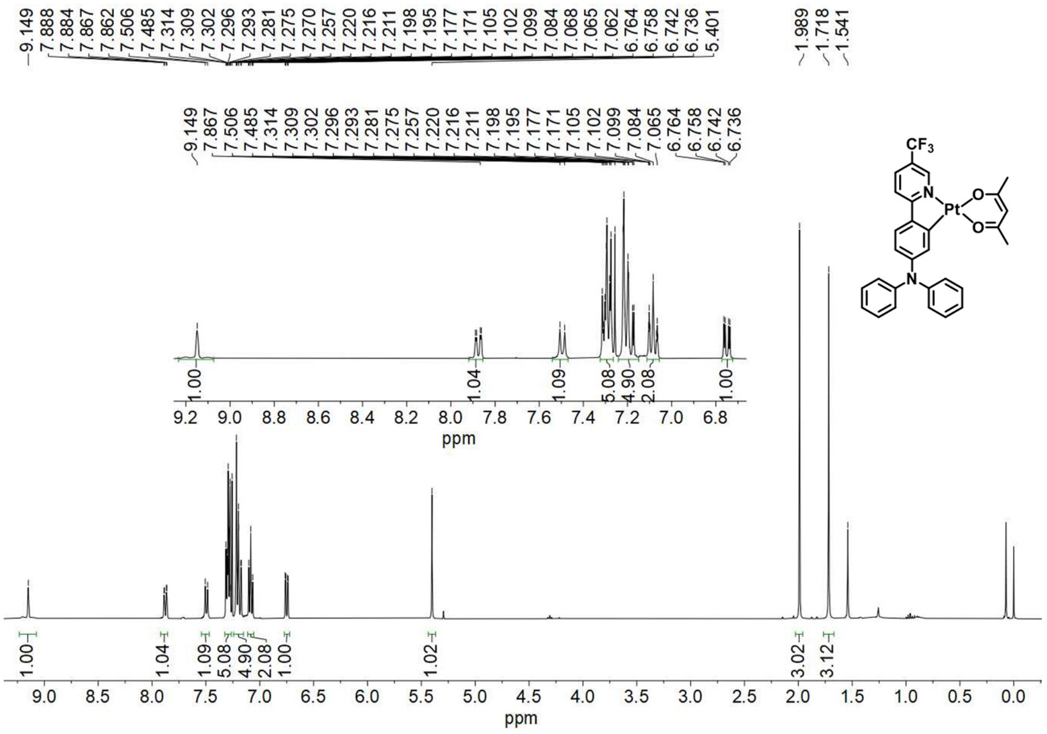

Figure S12. The ^{1}H NMR spectrum of **2** in $CDCl_3$.

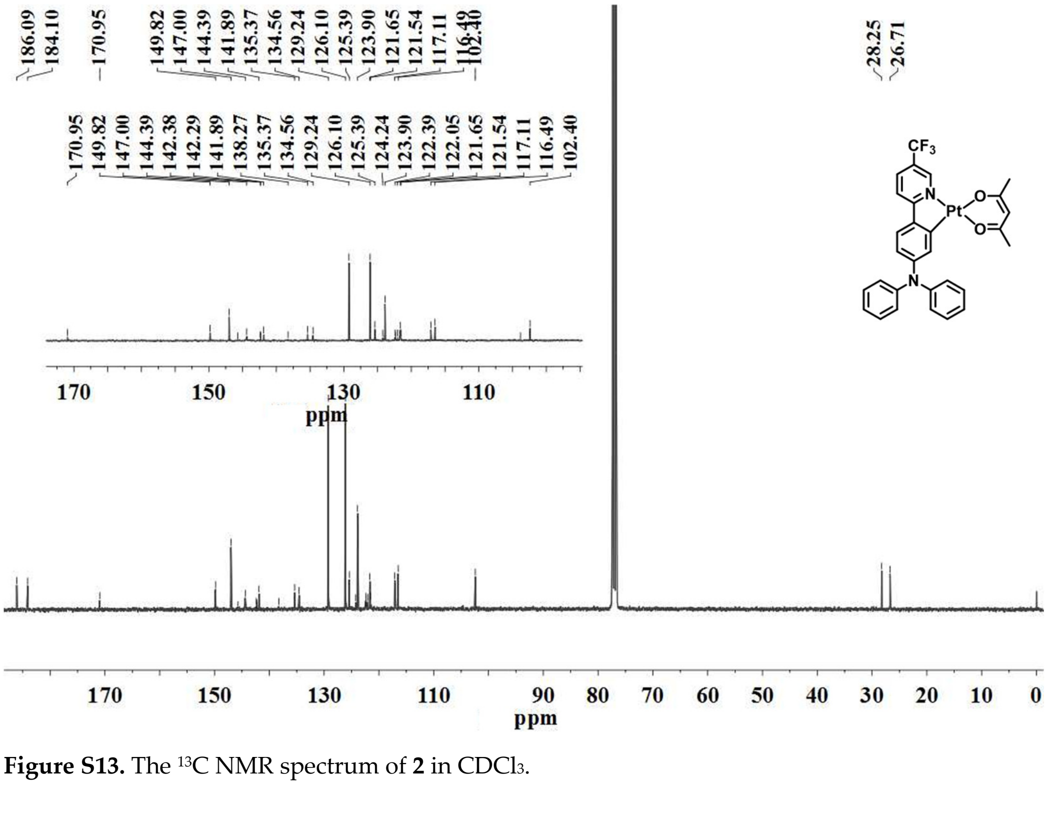

Figure S13. The ^{13}C NMR spectrum of **2** in $CDCl_3$.

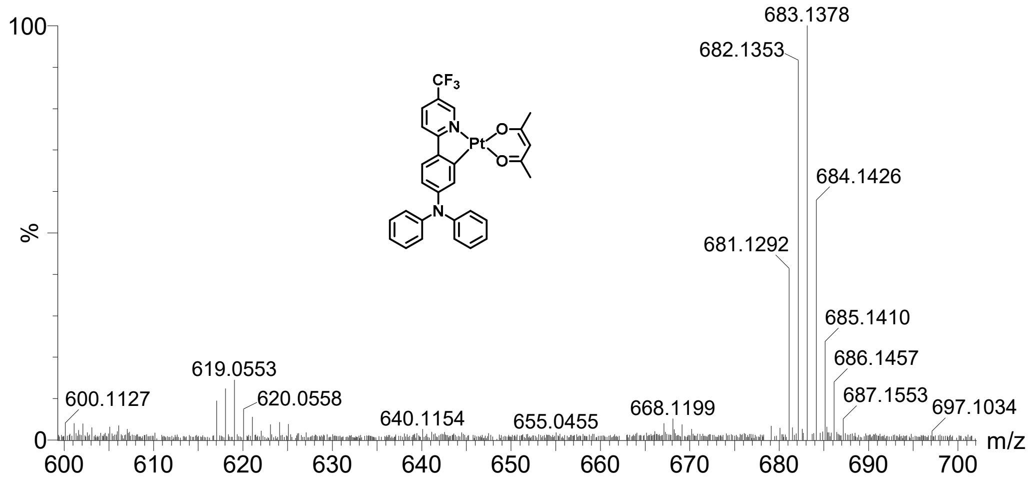

Figure S14. The HRMS of **2**.

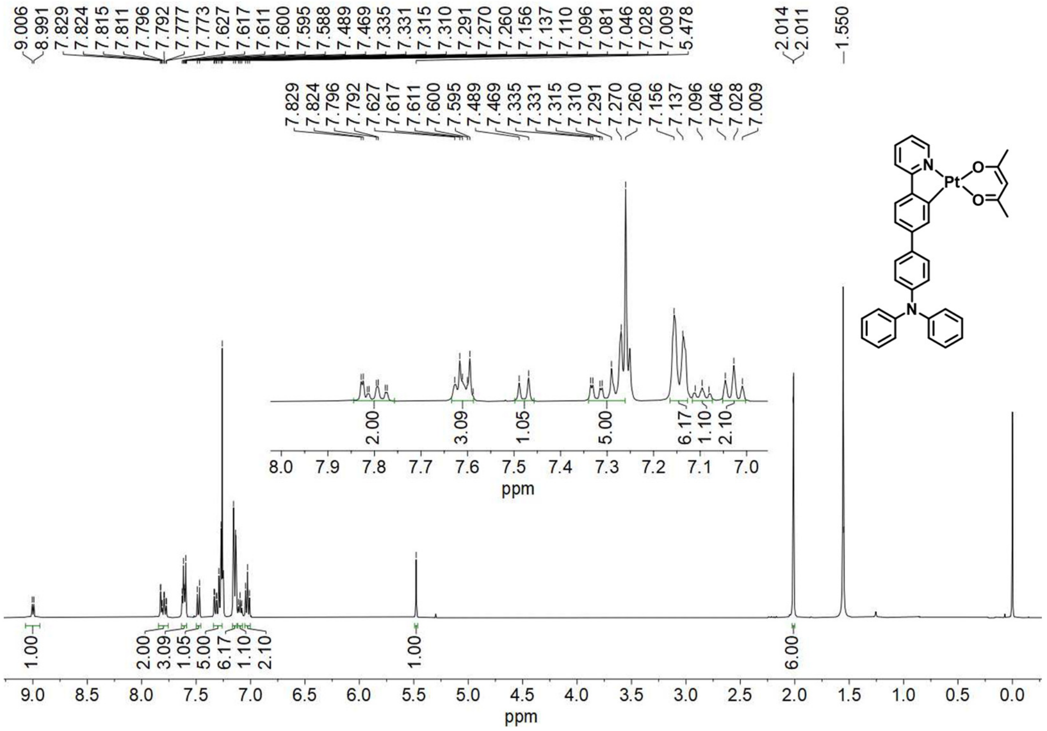

Figure S15. The ^{1}H NMR spectrum of **3** in $CDCl_3$.

References

[1] Yan, Y.; Jia, W.; Cai, R.; Liu, C. An AIPE-active fluorinated cationic Pt(II) complex for efficient detection of picric acid in aqueous media. *Chin. Chem. Lett.* **2024**, 35, 108819.

[2] Ghosh, S.; Mukherjee, P.S. Self-Assembly of a nanoscopic prism via a new organometallic Pt_3 acceptor and its fluorescent detection of nitroaromatics. *Organometallics* **2008**, 27, 316-319.

[3] Shanmugaraju, S.; Joshi, S.A.; Mukherjee, P.S. Self-assembly of metallamacrocycles using a dinuclear organometallic acceptor: synthesis, characterization, and sensing study. *Inorg. Chem.* **2011**, 50, 11736-11745.

[4] Shanmugaraju, S.; Samanta, D.; Gole, B.; Mukherjee, P.S. Coordination-driven self-assembly of 2D-metallamacrocycles using a shape-selective Pt^{II}_2-organometallic 90° acceptor: design, synthesis and sensing study. *Dalton Trans.* **2011**, 40, 12333-12341.

[5] Samanta, D.; Mukherjee, P.S. Pt^{II}_6 nanoscopic cages with an organometallic backbone as sensors for picric acid. *Dalton Trans.* **2013**, 42, 16784-16795.

[6] Maity, P.; Bhatt, A.; Agrawal, B.; Jana, A. Pt(II)C^N^N-based luminophore–micelle adducts for sensing nitroaromatic explosives. *Langmuir* **2017**, 33, 4291-4300.
[7] Hou, Y.; Shi, R.; Yuan, H.; Zhang, M. Highly emissive perylene diimide-based bowtie-shaped metallacycles. *Chin. Chem. Lett.* **2023**, 34, 107688.

Disclaimer/Publisher's Note: The statements, opinions and data contained in all publications are solely those of the individual author(s) and contributor(s) and not of MDPI and/or the editor(s). MDPI and/or the editor(s) disclaim responsibility for any injury to people or property resulting from any ideas, methods, instructions or products referred to in the content.
